# Long-Term Benefits of *Cenchrus fungigraminus* Residual Roots Improved the Quality and Microbial Diversity of Rhizosphere Sandy Soil through Cellulose Degradation in the Ulan Buh Desert, Northwest China

**DOI:** 10.3390/plants13050708

**Published:** 2024-03-01

**Authors:** Jing Li, Lili Zhang, Shikui Yu, Zongzhi Luo, Dewei Su, Dan Zheng, Hengyu Zhou, Jieyi Zhu, Xingsheng Lin, Hailing Luo, Christopher Rensing, Zhanxi Lin, Dongmei Lin

**Affiliations:** 1 National Engineering Research Center of Juncao Technology, Fujian Agriculture and Forestry University, Fuzhou 350002, China; fafulijing@fafu.edu.cn (J.L.); zhanglili@fafu.edu.cn (L.Z.); 13305913301@189.cn (S.Y.); luozongzhi@fafu.edu.cn (Z.L.); sudewei@fafu.edu.cn (D.S.); zhengdan@fafu.edu.cn (D.Z.); zhou.hengyu@outlook.com (H.Z.); 5220543016@fafu.edu.cn (J.Z.); singelin@163.com (X.L.); lhljuncao@fafu.edu.cn (H.L.); crensing94@gmail.com (C.R.); 2 College of Juncao and Ecology, Fujian Agriculture and Forestry University, Fuzhou 350002, China; 3 College of Life Science, Fujian Agriculture and Forestry University, Fuzhou 350002, China; 4 Institute of Environmental Microbiology, College of Resource and Environment, Fujian Agriculture and Forestry University, Fuzhou 350002, China

**Keywords:** long-term, *Cenchrus fungigraminus*, residual root, soil quality, soil microbial community

## Abstract

Long-term plant residue retention can effectively replenish soil quality and fertility. In this study, we collected rhizosphere soil from the residual roots of annual *Cenchrus fungigraminus* in the Ulan Buh Desert over the past 10 years. The area, depth, and length of these roots decreased over time. The cellulose content of the residual roots was significantly higher in the later 5 years (2018–2022) than the former 5 years (2013–2017), reaching its highest value in 2021. The lignin content of the residual roots did not differ across samples except in 2015 and reached its highest level in 2021. The total sugar of the residual roots in 2022 was 227.88 ± 30.69 mg·g^−1^, which was significantly higher than that in other years. Compared to the original sandy soil, the soil organic matter and soil microbial biomass carbon (SMBC) contents were 2.17–2.41 times and 31.52–35.58% higher in the later 3 years (2020–2022) and reached the highest values in 2020. The residual roots also significantly enhanced the soil carbon stocks from 2018–2022. Soil dehydrogenase, nitrogenase, and N-acetyl-β-D-glucosidase (S-NAG) were significantly affected from 2019–2022. The rhizosphere soil community richness and diversity of the bacterial and fungal communities significantly decreased with the duration of the residual roots in the sandy soil, and there was a significant difference for 10 years. *Streptomyces*, *Bacillus,* and *Sphigomonas* were the representative bacteria in the residual root rhizosphere soil, while *Agaricales and Panaeolus* were the enriched fungal genera. The distance-based redundancy analysis and partial least square path model results showed that the duration of residual roots in the sandy soil, S-NAG, and SMBC were the primary environmental characteristics that shaped the microbial community. These insights provide new ideas on how to foster the exploration of the use of annual herbaceous plants for sandy soil improvement in the future.

## 1. Introduction

Desertification is one of the important environmental and socioeconomic problems of desert scientific research and directly impacts the survival of human beings in the affected areas [1]. China is one of the countries most severely affected by desertification. According to the fifth survey of desertification in China, desertification caused by wind erosion affects 1.8 million km^2^, accounting for 69.9% of China’s desertified land area; these areas are mainly located in the northwestern, northern, and northeastern sandstorm areas of China. Due to continuous drought and cultivation in modern times, grasslands have been overgrazed, resulting in grassland degradation, shrinking rivers and lakes, and increasingly severe desertification. According to previous research, the Ulan Buh Desert has become one of the main sources of sand and dust that has negatively affected Chinese northern inland cities in recent years. Therefore, controlling the distribution and driving factors of desertified land in typical areas of northern China is very important [2]. The establishment of sand-fixing shrub plantations, which have been used extensively in northwestern China since the 1980s, is one of the most successful and sustainable measures to control desertification and restore degraded ecosystems [3]. Afforestation has been proposed to effectively promote soil development and enhance carbon sequestration by increasing net primary productivity and root biomass in sandy land [4]. Meanwhile, the studies emphasized stabilizing shifting dunes with native vegetation, increasing plant species richness and diversity, and improving soil nutrient content and organic matter [5,6,7]. Plant residual or plant litter retention strengthens biodiversity–ecosystem functioning relationships over time for uptake by improving plants, enhancing soil fertility, changing soil community composition, and reducing the impact of pathogens and pests [8,9]. According to Sandeep and Pritpal, rice residue retention significantly increased the water soluble-P concentration, carbon, and biological pools in soils under the rice–wheat cropping system [10].

*Cenchrus fungigraminus* Z. X. Lin & D. M. Lin & S. R. Lan sp. Nov. (Jujuncao) is a new species developed by Prof. Zhanxi Lin at Fujian Agriculture and Forestry University, China. Nowadays, *C. fungigraminus* is widely used in various fields, such as ecological management, mushroom cultivation, animal feed, and board production in China. Due to its developed root system and drought resistance ability [11], *C. fungigraminus* has been cultivated in Ningxia, Inner Mongolia, Xinjiang, Tibet, and Hainan since 2009 for sand fixation, water and soil preservation and high photosynthesis rates [12]. *C. fungigraminus* is a perennial plant in southern China, while in northern China, it is an annual plant, and temperatures of −5 °C can cause its death. Currently, the majority of studies have shown that the use of perennial, low-yield herbaceous plants in the deserts of China has significant effects on wind and sand fixation, enhancing soil quality and improving soil microbial structure [13,14,15,16]. However, since 2013, we have focused on planting high-yield, annual *C. fungigraminus* in the Ulan Buh Desert, and the aboveground part of *C. fungigraminus* has a significant effect on sand prevention and has generated substantial economic benefits as a feed or mushroom substrate. Yang et al. found that the increase in soil organic carbon (SOC) caused by rhizosphere sedimentation under root growth conditions was greater than that under root decomposition conditions after one year [17]. In that case, over the past ten years, many residual roots of *C. fungigraminus* have remained in sandy land. Does their degradation efficiency have a significant effect on improving organic matter, organic carbon, microbial carbon, enzyme activity, etc., in sandy land? Can residual root systems significantly enhance the microbial community and diversity in sandy land, thereby providing a reference for sandy land improvement in the long term?

The intensification of land desertification is one of the main ecological disasters, so it is necessary to strengthen agricultural scientific research in desertification areas. Therefore, exploring ways to increase the newly cultivated land and improving the arable land area and crop yield in the desertification area is beneficial to promote the sustainable development of agriculture in our country [18]. Soil is the largest carbon pool in terrestrial ecosystems, with over 80% of carbon stored in the soil [19]. The soil carbon stocks (SCS) are an important component of human life and natural ecosystems, and increasing carbon storage in sandy land can help protect the balance of ecosystems. Increasing soil organic matter (SOM) in soil is an important way to increase soil carbon storage and may greatly enhance the efficiency of fertilizer and water use and increase the resilience of agricultural systems to climate and environmental changes. Compared with traditional research on desertification control, we have used annual *C. fungigraminus* for wind prevention and sand fixation. However, after the aboveground parts were harvested, a large number of underground residual roots still exist in the sandy land for a long time. What effects will these residual roots have on the soil fertility and microbial structure of the rhizosphere sandy soil? In our study, to improve and maintain the quality of sandy land formed by *C. fungigraminus* residual roots and to achieve the diversification of sandy soil, it is necessary to understand the microstructure of sandy land to reflect soil fertility changes. The objectives of this study are to (1) reveal the inference of the changes in SOM, SOC, SCS, and soil microbial carbon composition (SMBC) in sandy soil over time; (2) determine the changes in the length and depth of the residual roots of *C. fungigraminus* over one decade; and (3) determine the changes in soil microbes in the rhizosphere of *C. fungigraminus* of different ages and their relationship with soil environmental factors. We hypothesized that the residual roots of *C. fungigraminus* continuously affected SOM and SOC in sandy rhizosphere soil over the past ten years, and simultaneously, the diversity of rhizosphere microbes significantly changed due to the influence of the residual roots of *C. fungigraminus.* This study can provide a theoretical basis for how the residual roots of *C. fungigraminus* promote the formation of sandy soil in the long term.

## 2. Material and Methods

### 2.1. Study Area Description

**Figure 1:**
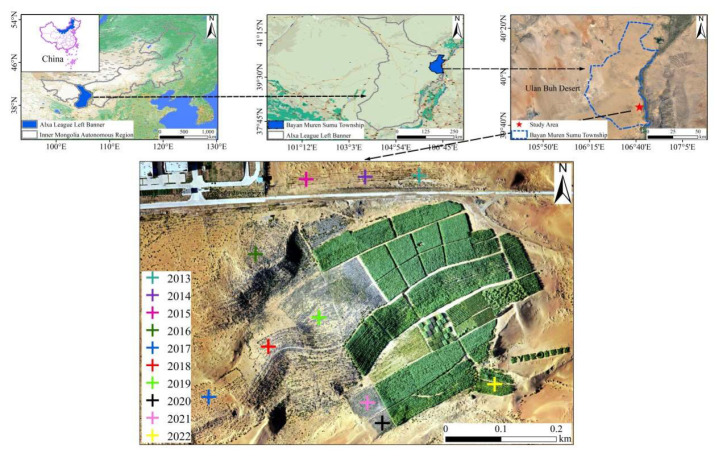
Study area and experimental field in the Ulan Buh Desert. A total of 10 years (from 2013 to 2022) of observations (different colors of “+”) were obtained in May 2023.

The research site is the National Engineering Research Center of Juncao Technology basement, which is located in the southern part of the Ulan Buh Desert in Alxa League of Inner Mongolia Province, China (106.702° E, 39.791° N) and is mostly composed of fixed and semifixed sand ridges and honeycomb-shaped sand dunes, with an average altitude of 1047.3 m (Figure 1). The area is typical arid and semiarid sandy land with an average annual temperature of 6–8.5 °C and an average annual precipitation of 90–215 mm. The total annual radiation is 4750–6250 MJ·m^−2^, the average annual sunshine duration is 3181–3500 h, and the frost-free period is 130–165 days. The sandy soil is aeolian sand developed from sandy parent material through wind action. The average annual wind speed is 3.1–4.6 m·s^−1^ and winds can blow gusts of sand and dirt in spring. The Ulan Buh Desert is one of the deserts in China with the richest water resources and little fluctuation in the aboveground terrain. The annual meteorological information in Ulan Buh Desert from 2013 to 2023 could be checked in https://www.worldweatheronline.com/ and http://data.cma.cn/.

### 2.2. Experimental Design and Field Sampling

We selected a typical sandy area where almost no vegetation types were present and the microtopography was fairly consistent and fenced with guardrail wire to prevent disturbance from animals and human activities. Each experimental plot was 25 m × 25 m, and *C. fungigraminus* was planted at 0.5 m (planting distance) × 1 m (line spacing) every April. Generally, basic fertilizer was applied once per year, and topdressing was applied 2 times. The basic fertilizer was 225 kg·hm^−2^ compound fertilizer and 225 kg·hm^−2^ diammonium phosphate, and the top application was 150 kg·hm^−2^ diammonium phosphate and 150 kg·hm^−2^ urea. The aboveground part of *C. fungigraminus* was harvested in October every year for silage, and the residual roots were retained in the sandy land from 2013 to 2022. All experimental areas were subjected to the same field management practices without any insecticides, pesticides, or animal grazing. We collected five residual roots in each experimental area and rhizosphere sandy soil of *C. fungigraminus* in May 2023. In addition, five rhizosphere soil samples were obtained from each healthy *C. fungigraminus* by gently shaking large soil blocks and collecting the soil that was tightly attached to the roots’ surface (approximately 1–20 mm). There were 10 treatments, each named T + Year (e.g., T2013); for example, T2013 means the residual roots were in the sandy soil for 10 years. The typical sandy land without any treatment was used as a control (CK) in this study. All soil and root samples were transported to the laboratory with dry ice. One part of the samples were stored at −20 °C for physical–chemical property testing within 48 h, while the remaining samples were stored at −80 °C in an ultralow temperature freezer (Thermo, Waltham, MA, USA) to perform high-throughput sequencing in the future.

### 2.3. Determination of the Physical–Chemical Properties of the Sand Soil

Sandy soil samples were ground to a smaller particle size and then sieved through a 2 mm mesh. The moisture of the samples was determined after drying in an oven (Jiangnan, China) at 65 °C. The soil bulk density method was according to Salehi Hikouei et al. [20]. The methods for determining SOM and SOC followed those of Yeomans and Bremner [21].

SCSs, including soil and biomass carbon, play a vital role in supporting human livelihoods and maintaining the balance of natural ecosystems. Increasing carbon storage in sandy soils can help safeguard ecosystem equilibrium and enhance agricultural productivity. In Equation (1) to Equation (3), *C_t_* represents the carbon stock of the land in year *t*, encompassing soil and residual root biomass; *C_SOC_*_,*t*_ represents the carbon stock in *SOC* in year *t* (*t* CO_2_·hm^−2^); *C_Root_*_,*t*_ represents the carbon stock in the residual root biomass in year *t*; *SOC_t_* represents the *SOC* content in year *t* (g·kg^−1^); *BD_t_* represents the soil bulk density in year *t* (g·cm^−3^); Depth represents the depth of the rhizosphere soil layer (cm); *CF_R_* represents the carbon fraction of dry matter from the residual roots; and *DM_R_* represents the dry matter (dm) mass of the residual roots per unit area (*t* dm·hm^−2^) [22,23].

(1) $$C_{t}=C_{soc,t}+C_{Root,t}$$

(2) $$C_{soc,t}=\frac{44}{12}\times SOC_{t}\times BD_{t}\times Depth\times 0.1$$

(3) $$C_{Root,t}=\frac{44}{12}\times CF_{R}\times DM_{R}$$

Soil dehydrogenase (S-DHA) catalyzes the oxidation–reduction reactions of sugars, organic acids, and amino acids. S-DHA activity was tested in the laboratory using a soil dehydrogenase kit (Solarbio, Beijing, China). Soil N-acetyl-β-D-glucosidase (S-NAG) catalyzes the breakdown of plant residues by microbes to produce nutrients, CO_2,_ and H_2_O, promoting the carbon cycle in soil and thereby increasing microbial diversity and soil fertility [24]. The activities of S-NAG and soil nitrogenase (S-NITS) were detected using an enzyme-linked immunosorbent assay (ELISA) kit (UPLC-MS, Shanghai, China). Soil microbial biomass, defined as the total mass of carbon in living and dead microorganisms in soil with a volume <5000 μm^3^, accounts for only 1% to 4% of the total soil organic carbon. SMBC was detected by the fumigation–extraction method [25,26].

### 2.4. Residual Root Measurement

We measured the root length and depth with steel tape. The residual root area was calculated by root length as a circle. Soil microbes often stabilize SOM by decomposing carbohydrates, cellulose, and lignin in plant residues [27]. Therefore, we determined the changes in total sugar (TS), cellulose, and lignin of the residual roots of *C. fungigraminus* using an ELISA kit (Molecular, Sunnyvale, CA, USA) to estimate the degradation of residual roots over ten years.

### 2.5. Total DNA Extraction and Sequencing

According to the physical–chemical properties of the sand soil and residual roots, we selected T2013 (long period), T2017 (middle period), and T2021 (short period) rhizosphere sandy soil, as well as CK, for bacterial and fungal high-throughput sequencing. The total genomic DNA of the residual root rhizosphere sandy soil was isolated by an E.Z.N.A.^®^ Soil DNA kit (Omega, Norcross, GA, USA), and the quality, concentration, and purity were detected by 1% agarose gels and a NanoDrop2000 (Thermo, Waltham, MA, USA). For bacterial community examination, the 16S rRNA gene primers with a barcode sequence were 338F (5′-ACTCCTACGGGAGGCAGCAG-3′) and 806R (5′-GGACTACHVGGGTWTCTAAT-3′). For sandy land fungal community determination, the internal transcribed spacer (ITS) gene region was amplified by ITS1F (5′-CTTGGTCATTTAGAGGAAGTAA-3′) and ITS2R (5′-GCTGCGTTCTTCATCGATGC-3′). The 16S rRNA gene V3–V4 variable region and ITS gene were amplified and purified using the NEXTFLEX ^®^ Rapid DNA-Seq kit (PerkinElmer, Shelton, CT, USA). The Illumina PE300 platform (Illumina, San Diego, CA, USA) was operated by Shanghai Majorbio Biopharm Technology Co., Ltd. (Shanghai, China) UPARSE v7.1 software (http://drive5.com/uparse/) [28,29], based on 97% similarity, and was used to perform operational taxonomic unit (OTU) clustering and for quality control of the spliced sequences and the removal of chimeras. Core OUTs (species) are the number of shared species among all samples, and are used to observe the decrease in the number of shared species as the sample size increases. The ribosomal database project (RDP) classifier [30] (http://rdp.cme.msu.edu/, version 2.11) was compared with the Silva 138 (bacterial) and Unite 8.0 (fungal) databases for OTU species taxonomy annotation; the confidence threshold was 70%, and the community composition of each sample was determined at different species classification levels. The 16S rRNA gene and ITS sequences derived from Illumina MiSeq data were deposited in the National Center for Biotechnology Information (NCBI) database (https://www.ncbi.nlm.nih.gov/) with the accession numbers PRJNA1002890 (accessed on 7 October 2023) and PRJNA1004229 (accessed on 11 October 2023).

### 2.6. Bioinformatic Processing and Statistical Analysis

All sequence data analysis was conducted on the Major Biocloud platform (https://cloud.majorbio.com). Alpha diversity and difference analysis were performed using Mothur software (version 1.30.2) (http://www.mothur.org/wiki/Calculators) [31] and the Wilcoxon test. Principal coordinate analysis (PCoA) based on the Bray–Curtis distance algorithm was used to test the similarity of the microbial community structure between samples and was combined with the PERMANOVA nonparametric test to analyze whether the differences in the microbial community structure between sample groups were significant. Linear discriminant analysis effect size (LEfSe) (http://huttenhower.sph.harvard.edu/LEfSe) (LDA > 2, *p* < 0.05) was used to identify bacterial or fungal groups with significant differences in abundance from the phylum to the genus level among different groups [32]. R studio 4.3.1 (Rstudio, Michigan, MI, USA) and pheatmap 1.0.8 were used for community species composition and species abundance in hierarchical clustering analysis (average linkage method) and heatmap structure. The co-occurrence network was analyzed using Networkx (1.11) (Python, Netherland). Distance-based redundancy analysis (db-RDA) was used to investigate the effects of soil physicochemical indicators on the structures of the soil bacterial and fungal communities according to canonical analysis of principal coordinates (CAP). Species were selected based on Spearman’s correlation |r| > 0.6, *p* < 0.05 for correlation network diagram analysis [33]. Functional prediction analysis was performed using PICRUSt2 (version 2.2.0) software [34]. R studio 4.3.1 was used to construct partial least square path models (PLS-PM) for the soil physical and chemical properties, microbial abundance, and enzyme activities in different treatments. SPSS 26.0 (IBM, Armonk, NY, USA) and Excel (Microsoft, Redmond, WA, USA) were used for statistical analysis. The normality test was carried out to ensure that the experimental data followed a normal distribution. We used analysis of variance (ANOVA) to test for significant differences among the treatments. A least significant difference (LSD) test was performed to determine any differences among the treatments. Significance was calculated by Duncan’s test (*p* < 0.05). Values followed by lowercase letters were significantly different (*p* < 0.05). All graphs were drawn using Photoshop 2020, Adobe Illustrator 2022 software (Adobe, San Jose, CA, USA), and GraphPad°Prism 8.0 software (GraphPad, Santiago, CA, USA).

## 3. Results

### 3.1. Sandy Soil and C. fungigraminus Residual Root Measurements

According to Table 1, as the time of *C. fungigraminus* residual roots in the sandy land increased, the rhizosphere soil bulk density gradually increased. The SOM and SOC decreased briefly but then increased overall and reached the highest values in T2020. The SOM content was 2.17–2.41 times higher and the SMBC content was 31.52–35.58% higher in T2020-T2022 than in CK. Meanwhile, the SOM content was 1.45–1.76 times higher and aSMBC was 12.11–19.61% higher in T2013-T2015 than in CK. From T2020 to T2022, the SMBC in all treatments was significantly higher than that from T2013 to T2019 (*p* < 0.05), and the same was true for S-NITS. Before 2018, S-DHA activities decreased over time. S-NAG significantly increased in the *C. fungigraminus* residual root treatment compared to CK (*p* < 0.05) and increased overall. From T2018 to T2022, there was a significant difference in the carbon stock in the rhizosphere soil of the residual root system. As the time of the residual roots increased in the soil, the root depth decreased year by year, and the carbon stock in the rhizosphere soil reached its lowest level in T2013.

**Figure 2:**
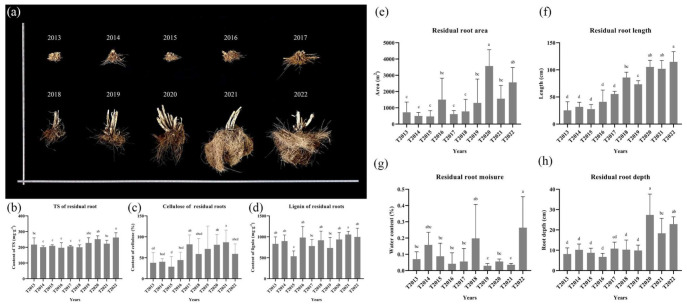
The residual roots over the past ten years (**a**), TS (**b**), cellulose (**c**), and lignin (**d**) contents of residual roots and their physical properties (**e**) is the residual root area, (**f**) is the residual root length, (**g**) is the residual root moisture, and (**h**) is the residual root depth). Note: Total sugar (TS). The different lowercase letters were shown significantly different (*p* < 0.05).

In Figure 2, the area, depth, and length of *C. fungigraminus* residual roots decreased as time elapsed. However, the moisture of all soil samples was without any difference with CK (0.21 ± 0.18%). The cellulose content of *C. fungigraminus* residual roots was significantly higher from T2018 to T2022 and reached its highest value in T2021 (86.93 ± 29.22%). Both the TS and lignin of *C. fungigraminus* residual roots were mostly stable over the 10-year period. The lignin among all samples, except in T2015, was not different and reached the highest level in T2021 (906.23 ± 78.68 mg·g^−1^). The TS in T2022 was 227.88 ± 30.69 mg·g^−1^ and was significantly higher than those in the other treatments. 

### 3.2. Basic Sequencing Data of the Four Sandy Soils

DNA was extracted from four treatments (CK, T2013, T2017, and T2021) of *C. fungigraminus* residual root rhizosphere soil samples. High-throughput sequencing analysis was based on 16S rRNA and ITS gene sequencing from the Illumina PE300 platform. Based on Silva (Release 138 http://www.arb-silva.de) and Unite (Release 8.0 http://unite.ut.ee/index.php), a total of 1,729,208 quality-filtered and chimera-checked 16S rRNA sequences were obtained with an average length of 415 bp across all samples. These included 1 domain, 1 kingdom, 37 phyla, 129 classes, 334 orders, 563 families, 1157 genera, and 2503 species. Meanwhile, a total of 1,685,026 quality-filtered and chimera-checked ITS gene sequences were obtained with an average length of 242 bp across all samples, which included 1 domain, 1 kingdom, 11 phyla, 30 classes, 59 orders, 121 families, 221 genera, and 364 species. In total, based on 97% sequence similarity, there were 9670 OTUs in the bacterial communities and 908 OTUs in the fungal communities. All core OTUs were T2021 > T2017 > T2013 > CK (Figure S1a,b). The rarefaction curve tended to be flat, indicating that the sequencing data were acceptable and that the sequence depth was sufficient (Figure S1c,d).

Alpha diversity indices, including bacterial and fungal community richness (Sobs, Chao, and ACE), evenness (Shannoneven), diversity (Shannon), and coverage index values, were compared for different groups (Table 2). The Chao, ACE, and Sobs estimators indicated that the bacterial and fungal community abundances in T2021 were significantly higher than those in the other groups. The Shannon indices of the bacterial and fungal communities showed that the microbial communities became significantly more diverse over time. However, there was no significant difference in bacterial abundance and coverage between T2013 and T2017. All treatments had good community coverage and shannoneven values. Nonmetric multidimensional scaling (NMDS) was performed using the Bray–Curtis method for beta diversity clustering, and an Adonis difference test analysis was conducted on the microbial composition of all samples. We obtained an NMDS model with R^2^ = 0.833, *p* < 0.01 (bacterial) and R^2^ = 0.737, *p* < 0.01 (fungal). The residual root system of *C. fungigraminus* had a greater impact on the bacterial community composition than the fungal community composition from T2013 to T2021. Compared to T2021, T2013 and T2017 had a more similar bacterial community composition; however, in terms of fungal composition, T2013 was closer to T2021 (Figure S2a,b).

### 3.3. Taxonomic Profile of Sandy Soil Bacterial and Fungal Communities

**Figure 3:**
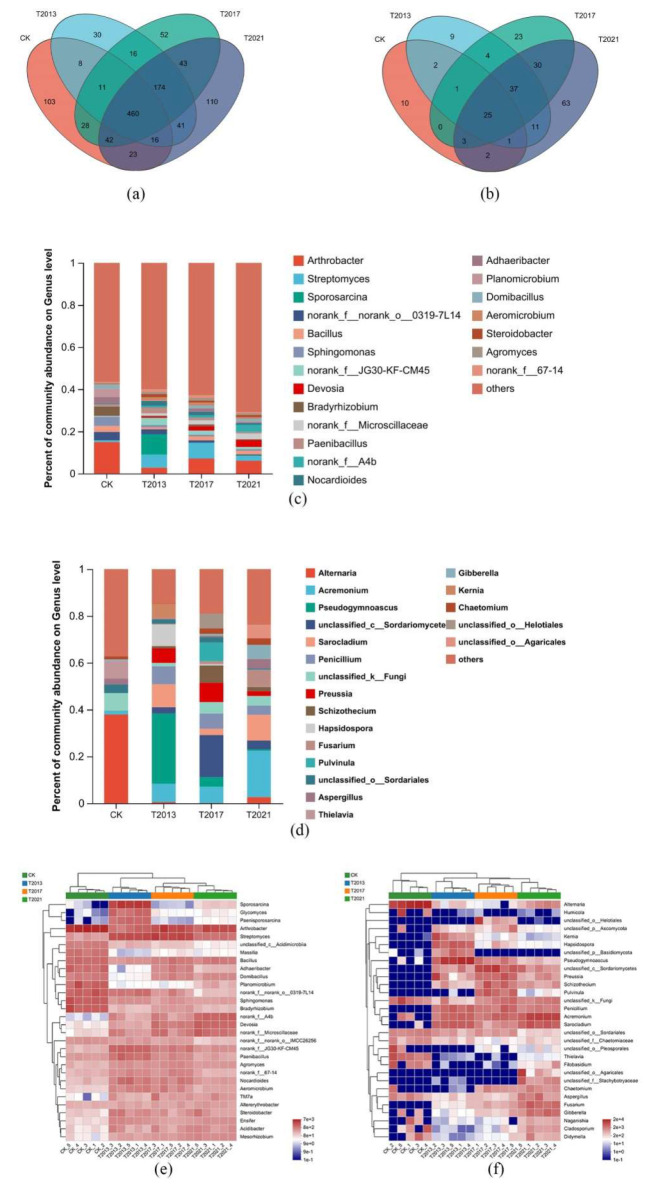
Venn plots of the *C. fungigraminus* residual roots’ rhizosphere sandy soil bacteria (**a**) and fungi (**b**) at the genus level in the four treatments. Relative abundance of the residual root rhizosphere sandy soil bacterial (**c**) and fungal (**d**) community analysis (top 20) at the genus level in the four treatments. Heatmap analysis of the *C. fungigraminus* residual roots’ rhizosphere sandy soil bacterial (**e**) and fungal (**f**) composition at the genus level in the four treatments (top 30) and hierarchical clustering analysis based on the average linkage method.

To further examine the bacterial community structure in the rhizosphere sandy soil of the residual roots of *C. fungigraminus*, taxonomic classification at the phylum and genus levels was performed, as shown in Figure 3a,c and Figure S3a,c. Among the four treatments, the main and mutual phyla included Actinobacteria (the relative abundance was 19.81–31.87%), Proteobacteria (19.59–33.93%), Firmicutes (5.65–17.54%), Bacteroidetes (6.08–12.32%), Chloroflexi (5.87–9.88%), and Patescibacteria (0.1–4.25%). There were 30 different phyla, including Spirochaetota (the average relative abundance was 73.68%) and NB1-j (26.32%), only in CK. There were 460 different genera among all samples, and the most common genus was *Arthrobacter* (8.13%), followed by *Streptomyces* (4.46%), *Sporosarcina* (2.64%), 0319-7L14 (2.01%), *Bacillus* (1.99%), *Sphingomonas* (1.86%), JG30-KF-CM45 (1.80%), *Devosia* (1.61%), *Bradyrhizobium* (1.60%), and *Microscillaceae* (1.60%). *Streptococcus* (4.18%), *Kurthia* (2.86%), *DSSF69* (2.42%), *Bacteroides* (2.31%), *Clostridia*_UCG-014 (2.31%), *Ktedonobacteraceae* (2.31%), *Proteiniborus* (2.20%), and *Keratinibaculum* (2.09%) were found only in CK. In T2013, *Phytoactinopolyspora* (44.03%), AKAU4049 (18.28%), SHA-26 (5.97%), *Chlamydiaceae* (3.36%), *Sandaracinaceae* (3.36%), *Nocardioidaceae* (2.61%), *Salipaludibacillus* (2.24%), and *Methyloligellaceae* (2.24%) were unique. Meanwhile, the endemic genera in T2017 included *Cystobacter* (57.46%), *Eubacterium* (8.25%), *Anaerococcus* (5.24%), *Desulfovibrio* (5.24%), and *Soehngenia* (3.14%). In T2021, the only bacterial genera were *Emticicia* (17.71%), *Rhodanobacter* (7.08%), *Arachidicoccus* (3.85%), *Desemzia* (3.75%), *Rhodocyclaceae* (3.23%), *Smaragdicoccus* (3.02%), *Parafilimonas* (2.71%), *Luteibacter* (2.60%), and *Oscillochloris* (2.60%) (Figure 3e and Figure S3e). Regarding the different microbial structures, the relative abundance of *Arthrobacter* was 14.93%, while in the *C. fungigraminus* residual roots in sandy soil, the abundance was reduced to 2.78–7.01%; *Streptomyces* abundance reached a maximum of 7.57% in T2017. Moreover, the abundance of *Sporosarcina* was higher, at 9.72%, than that of the other groups. From T2013 to T2021, the abundance of *Sphingomonas* ranged from 0.8% to 1.1%, while the abundance reached 4.31% in CK. According to the Kruskal–Wallis and Tukey–Kramer tests, *Arthrobacter, Streptomyces*, and *Sporosarcina* were significantly different among all treatments (*p* < 0.05). With the duration of the residual roots of *C. fungigraminus* in the sandy land, the abundances of *Devosia*, *Microscillaceae*, JG30-KF-CM45, *Paenibacillus*, and *Nocardioides* increased by 1.27 to 5.87 times compared with those in CK.

Regarding the fungal community OTUs, as shown in Figure 3b and Figure S3b, the main phyla were Ascomycota (83.70–90.97%), Basidiomycota (3.23–11.51%), Mortierellomycota (0.1–1.36%), and Chytridiomycota (0.2–1.16%). Among these, Ascomycota, Basidiomycota, and Chytridiomycota were the common phyla. In T2017, the endemic phylum was Calcarisporiellomycota. In T2021, the endemic phyla were Aphelidiomycota (10.00%) and Kickxellomycota (90.00%). At the genus level, the common fungi included *Altermaria* (17.37%), *Acremonium* (15.19%), *Pseudogymnoascus* (14.72%), *Sarocladium* (9.99%), and others. In CK, the only communities were *Neodidymeliopsis* (32.10%), *Neocucurbitaria* (22.61%), *Camarosporidiellla* (16.83%), *Thermomyces* (11.46%), and *Coniophora* (5.13%). The endemic genera in T2013 included *Rhizophlyctis* (64.61%), *Paracylindrocarpon* (12.92), *Tremellales* (6.74), and *Powellomycetaceae* (5.62%). *Cephaliophora* (23.67%), *Spegazzinia* (17.54%), *Nematoctonus* (12.82%), *Entoloma* (12.47%), *Mallocybe* (7.50%), *Chordomyces* (5.58%), *Leohumicola* (5.58%), and *Neosulcatispora* (3.80%) were found only in T2017. *Agaricales* (66.31%), *Panaeolus* (20.32%), *Curvularia* (3.54%), and *Rhodotorula* (1.75%) were present only in T2021 (Figure 3d and Figure S3f). In Figure 3d,f, *Acremonium* (6.99–19.80%) was present in T2013, T2017, and T2021 but not in CK (1.43%). Furthermore, *Preussia* (2.00–8.26%) was present in T2013, T2017, and T2021, but not in CK. However, *Alternaria* (37.91%) was significantly higher in CK than in the other groups (0.52–2.62%). Based on the Kruskal–Wallis and Tukey–Kramer tests, *Alternaria* and *Acremonium* were enriched genera in CK and other treatments, respectively (*p* < 0.05).

### 3.4. Microbial Communities, Functional, and Co-Occurrence Network Analysis

The LEFse tool can analyze microbial community data in any clade. We analyzed the bacterial and fungal OTUs from the domain to the genus level. The results are shown in Figure S4a, and the LDA score for bacteria was 3.5. In CK, *Planomicrobium*, *Bradyrhizobium*, *Sphingomonas*, *Adhaeribacter*, *Massilia*, and *Herbaspirillum* were enriched and made the primary contributions to the majority. In T2013, *Sporosarcina*, JG30-KF-CM45, *Paenisporosarcina*, *Glycomyces*, *Paenibacillus,* and *Nocardioides* were enriched. In T2017, *Streptomyces*, f_67-14, *Hyphomicrobium*, *Agromyces*, and *Dongia* were enriched. In T2021, f_A4b, *Devosia*, *Microscillaceae*, f_BIrii41, *Mesorhizobium*, and *Acidibacter* were enriched. The LDA score for fungi was 2, and *Alternaria*, *Thielavia*, *Filobasidium*, *Sordariales*, and *Cladosporium* were enriched in CK. In T2013, *Pseudogymnoascus*, *Hapsidospora*, *Penicillium*, *Kernia*, *Wardomyces*, and *Mortierella* were the primary genera and were enriched. In T2017, *Sordariomycetes*, *Pulvinula*, *Preussia*, *Schizothecium*, *Lasiosphaeriaceae*, *Stachybotrys*, and *Cyphellophora* were enriched. In T2021, *Acremonium*, *Sarocladium*, *Fusarium*, *Gibberella*, *Aspergillus*, *Chaetomium*, *Hypocreales*, *Apiospora*, etc., were enriched (Figure S4b). Through network analysis, we explored the interaction effects of residual roots in sandy land over different periods of time on the soil bacterial and fungal communities. The difference in the co-occurrence networks at the genus level among all samples revealed that the connectivity, degree centrality, compactness coefficient, and betweenness centrality of the bacterial co-occurrence network in CK, T2013, and T2017 were lower than those in T2021. The network topology characteristics (connectivity, degree centrality, compactness coefficient, betweenness centrality) of fungi in T2021 were higher than those in CK, T2013, and T2017 (Table 3, Figure S4c,d). Therefore, the extended duration of the *C. fungigraminus* residual root system in sandy land may lead to an increase in the symbiotic relationship between microbial taxa.

**Figure 4:**
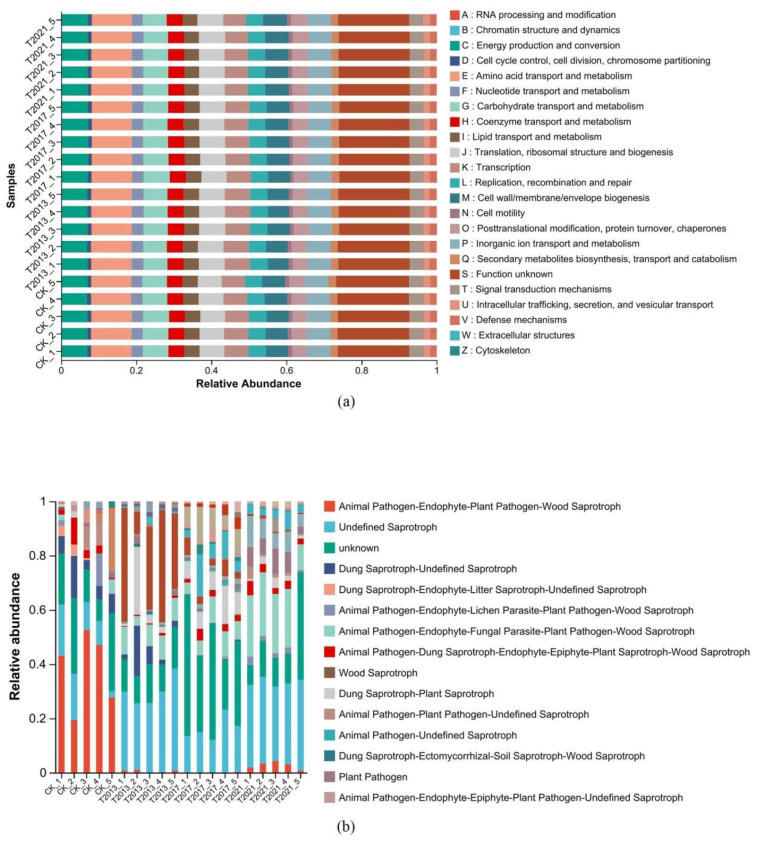
COG function classification of the residual root rhizosphere sandy soil bacterial community (**a**) and fungal community functional classification by FUNGuild (**b**) (the relative abundance decreases sequentially from top to bottom).

According to PICRUSt2, we predicted the bacterial function (Figure 4a); the functions were mainly energy production and conversion, amino acid transport and metabolism, carbohydrate transport and metabolism, coenzyme transport and metabolism, replication, recombination, and repair. Moreover, based on FUNGuild, the most important pathways were the animal pathogen-wood saprotroph, animal pathogen-endophyte-fungal parasite-plant pathogen-wood saprotroph, plant pathogen, and animal pathogen-endophyte-lichen parasite-plant pathogen-soil saprotroph-wood saprotroph (Figure 4b).

### 3.5. Correlations between Bacterial and Fungal Structures and Environmental Variables

**Figure 5:**
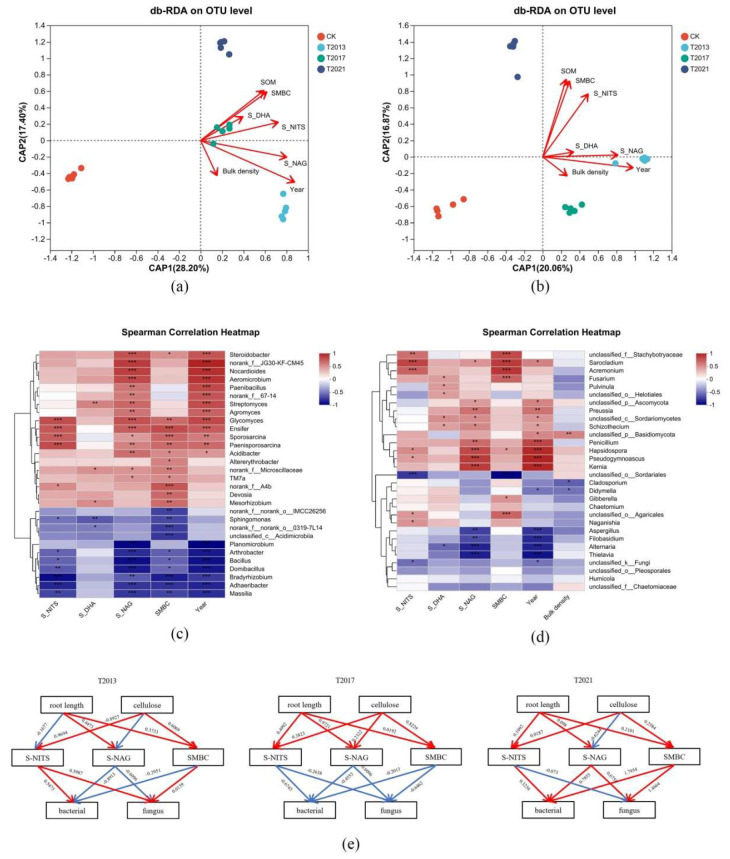
db-RDA of the *C. fungigraminus* residual roots’ rhizosphere sandy soil bacterial (**a**) and fungal (**b**) OTUs and environmental factors; Spearman correlation heatmap of the residual root rhizosphere sandy soil bacterial (**c**) and fungal (**d**) species at the genus level between the main environmental factors (the correlation coefficient between environmental factors and selected species is represented by the size of data values through color from red to blue, with red indicating strong correlation and blue indicating the opposite); (**e**) Direct associations among variance inflation factor including root length, cellulose, SMBC, S-NITS, S-NAG, and microbial abundance (the blue arrow indicates negative effect and red one indicates positive effect). Note: Soil organic matter (SOM), soil microbial biomass carbon (SMBC), soil dehydrogenase (S-DHA), N-acetyl-β-D-glucosidase (S-NAG), and soil nitrogenase (S-NITS). * were significantly different (*p* < 0.05), ** were highly significantly different (*p* < 0.01) and *** were extremely significant difference (*p* ≤ 0.001).

The different durations of *C. fungigraminus* residual roots in rhizosphere sandy land had different effects on the soil environmental characteristics and changed the microbial community structures. The effect of rhizosphere sandy land microbial communities may primarily be mediated by soil physical–chemical characteristics and the duration of the residual roots. Therefore, we investigated whether the environmental factors and microbial structure were related. We screened all the environmental factors through the variance inflation factor (VIF), and the results showed that S-NITS (VIF is 5.44) > SMBC (4.75) > S-NAG (3.08) > Year (3.14) > bulk density (1.21) > S-DHA (1.19). Based on Figure 5a,b, the duration of the *C. fungigraminus* residual roots in the sandy land (Year), S-NAG, and SMBC were the primary environmental characteristics that affected the bacterial community, while the duration of the *C. fungigraminus* residual roots in the sandy land (Year), S-NAG, S-NITs, and SMBC were the main environmental factors that affected the fungal community; however, they had a negative influence on CK. The Spearman correlation heatmap showed that the duration of the *C. fungigraminus* residual roots in the sandy land (Year) and S-NA had a positive effect on *Steroidobacter*, JG30-KF-CM45, *Nocardioides*, *Aeromicrobium*, *Paenibacillus*, f_67-14, *Streptomyces*, *Agromyces*, *Sporosarcina*, *Paenisporosarcina*, *Ensifer,* and *Glycomyces*; SMBC had a positive influence on *Sporosarcina*, *Paenisporosarcina*, *Ensifer*, *Glycomyces*, *Acidibacter*, *Microscillaceae*, TM7a, *Altererythrobacter*, f_A4b, *Devosia,* and *Mesorhizobium* (*p* < 0.05); while S-NITS had a positive influence only on *Sporosarcina, Paenisporosarcina*, *Ensifer*, and *Glycomyces* (*p* < 0.05). The environmental factors except S-DHA had significantly negative effects on *Arthrobacter*, *Bacillus*, *Domibacillus*, *Bradyrhizobium*, *Adhaeribacter*, and *Massilia* (*p* < 0.05) (Figure 5c). The Spearman correlation showed that year and S-NAG had a positive influence on *Sarocladium*, *Acremonium*, *Ascomycota*, *Preussia*, *Sordariomycetes*, *Schizothecium*, *Penicillium*, *Hapsidospora*, *Pseudogymnoascus,* and *Kemia* but had a negative effect on *Aspergillus*, *Filobasidium*, *Alternaria*, and *Thielavia* (*p* < 0.05, Figure 5d).

The PLS-PM has been widely applied to study the complex multivariate relationships between variables, this study uses this model to infer the potential indirect effects of major factors such as root length, cellulose, S-NITS, S-NAG, and SMBC on bacterial and fungal abundance. The PLS-PM results of goodness of fit (GOF) values in T2013, T2017, and T2021 were 0.582, 0.543, and 0.452, respectively. According to Figure 5e, different retention times of residual roots alter soil environmental characteristics and microbial community abundance. The decomposition of plant root cellulose and root length has a positive effect on the accumulation of SMBC; the decomposition of roots has a positive effect on S-NITS and S-NAG activities. In T2013 and T2021, S-NITS and S-NAG activities in residual roots can promote the diversity of bacteria and fungi in soil.

## 4. Discussion

Environmental protection policies, along with human intervention and the control of desertification, have played important roles in reversing desertification. From 2010 to 2020, under the general background of a warm-wet climatic tendency, the rational use of sand resources and strengthening the scientific control of desertification effects of plant residues on soil physicochemical properties and carbon sequestrationication-inducing factors were the keys to reversing desertification [2]. Compared with traditional perennial desert plants, *C. fungigraminus* has the characteristics of annual growth, developed root systems, and high biomass in northern desertification areas. After natural apoptosis, a large number of plant roots still remain in the sandy land, and these residual roots can exist for up to 10 years or even longer and the impact on sandy soil is significant.

### 4.1. Effects of Plant Residue Retention on Soil Physicochemical Properties and Carbon Stock

Plant residues or litter include the aboveground parts (leaves and stems) and underground parts (residual roots and root exudates) of plants. Plant residues affect soil organic matter accumulation by directly affecting organic matter input and indirectly affecting microbiology to realize the plant residues interact with soil ecosystems [35]. On the one hand, plant residues directly form soil particulate organic carbon through physical or chemical decomposition [36]; On the other hand, plant residues circulate through microbial carbon pumps and combine with minerals in the soil to form mineral-bound organic carbon [37]; meanwhile, plant residues are also one of the carbon sources for microbial metabolism, affecting soil microbial carbon utilization efficiency and microbial residual carbon [38]. The input of plant residues caused by human interference is a common phenomenon in terrestrial ecosystems, which plays an important role in regulating the nutrient level of terrestrial ecosystems. Plant residue input is not only one of the soil carbon sequestration measures but is also a potential technology for achieving a global soil carbon increase plan of 4‰ [39]. In addition, there are complex interactions between soil microbial and environmental factors that dominate the soil carbon and nitrogen cycles, and SOM is important for sustainable agricultural production. The plant residues significantly increased SOC by 13.98% after one year; there was increased accumulation of the fungal community under low soil fertility, and high fertility promoted the accumulation of the bacterial community [40]. The contribution of corn roots to microbial phospholipid-derived fatty acids was significantly higher than the stems and leaves; corn roots were also shown to increase the contribution of microbial residues to the soil carbon pool [41,42]. When apple branches were returned to the field for 3 years, the cellulose content was significantly reduced, the molecular structure of lignin largely disintegrated, and chemical bonds were broken [43].

According to the previous study, the SOC and SOM sequestration occurs through the destruction of lignocellulose components by microbial activities [13,15,44]. The SOM in all size aggregates in the rhizosphere soil of both coarse and tap roots was higher than that of the nonrhizosphere soil [44]. SOC in tropical savannas mostly originates from grasses because both forest-floor litter and roots are known to be involved in the formation of SOC, and there are different roles and contributions of forest-floor litter and roots to the pathways of SOC formation and the spatial differentiation of carbon compounds [8,45]. Between 1982 and 1999, there was a significant increase in the aboveground biomass of grasslands in China, demonstrating strong carbon sinks (17.7 Tg C year^−1^) [46]. However, under the dual interference of climate change and human activities, 70–90% of grasslands in China have undergone varying degrees of degradation, resulting in significant carbon loss before 2010 [47]. Soil is the largest organic carbon pool in terrestrial ecosystems, with an organic carbon content of approximately 1500 Gt in soil at a depth of 1 m worldwide, exceeding the total organic carbon storage of vegetation and the atmosphere. The composition of the soil organic carbon pool includes plant, animal, and microbial residues, excreta, secretions, and their decomposition products, including soil humus. Soil organic carbon plays an extremely important role in the global carbon cycle process [48]. Xin et al. found that organic carbon in 0–100 cm soil was lost at a rate of 48.4 Tg C year^−1^ between 1963 and 2007 [49]. There is a significant positive correlation between plant root traits (root biomass, underground plant carbon, nitrogen, and phosphorus content) and microbial residual carbon [50]. According to He et al., the amount of plant carbon input is significantly positively correlated with the content of microbial residual carbon in soil, and compared to deep soil, the surface layer experiences more variation [51]. In a 30-year recovery series of temperate grasslands, underground plant biomass significantly affects the accumulation of plant and microbial residual carbon [52]. The physical and chemical properties of soil, such as moisture, pH, bulk density, soil C/N, SOC, and soil aeration conditions, also have a significant impact on the formation and stability of plant and microbial residual carbon, and are another important driving factor after the climate and environment [53,54]. In this study, over the span of ten years, the SOM, SOC, and SCS of rhizosphere soil decreased over time and reached the highest levels in 2020, as did S-NAG and S-NITS. Crop diversity-driven changes in root traits induced microbial traits and promoted microbial necromass accumulation. Compared with bacterial residues in the rhizosphere of rice, fungal necrotic groups are less likely to decompose and are the largest contributors to carbon sequestration through mineral-related organic carbon components and root sediments [55]. Our results also showed that the residual roots of *C. fungigraminus* can increase SCS in sandy land, while it has significantly enhanced in the short-term (2018–2022). Moreover, the *C. fungigraminus* residual root area, depth, and length decreased over time. The lignin content of the *C. fungigraminus* residual roots was higher in the short term (2018–2022) than in the long term (2013–2017) and reached the peak value in T2021. The cellulose content of the *C. fungigraminus* residual roots was significantly higher in the short term (2018–2022) than the long term (2013–2017). The TS of the *C. fungigraminus* residual roots in T2022 was significantly higher than that in other years. The correlation between plant biodiversity, soil fertility, and productivity was reported to become stronger over time in grasslands, forests, deserts, and agroecosystems. Plant shoot and root litter were important in mediating these positive correlations, but the functional role of residual roots remains overlooked in long-term experiments [9]. We propose that plant residual roots strengthen soil quality through the decomposition of residual root cellulose, which releases nutrients over time for uptake by existing and succeeding plants and enhances overall soil fertility.

### 4.2. Effect of Plant Residues on Soil Microbial Structure and Diversity

In the rhizosphere soil, plants alter the physicochemical properties and modify and restrict the microbial community of rhizosphere soil through root exudates such as carbohydrates, amino sugars, organic acids, vitamins, and fatty acids. Soil microbial diversity can reflect the changes in soil quality early and reveal differences in microbial ecological functions, and is considered one of the most promising sensitive biological indicators [56]. The activities and interactions of the microbial community greatly affect soil productivity and nutrient cycling, as well as other ecosystem characteristics and processes [57]. The microbiome stabilization mediated by the host plant was shown to enable the consistent retention of beneficial bacteria (Actinobacteria and Alphaproteobacteria) with multiple plant growth-promoting functions, including those capable of producing extracellular polymeric substances, which increase the water-holding capacity of sand in microenvironments [58]. In our study, *Streptomyces, Bacillus,* and *Sphingomonas* were the main bacterial genera of the microbial community. *Streptomyces* has been shown to promote plant growth, enhance nutrient absorption, and enhance resistance to biotic and abiotic stresses [59]. *Streptomyces lydicus* promoted tomato growth, increased jasmonic acid, salicylic acid, and other growth regulators, and enhanced photosynthesis [60]. *Streptomyces* also increased the carbon and phosphorus contents in chickpeas [61]. *Streptomyces* secretes antibacterial compounds that also play a significant role in plant disease resistance [62,63]. According to previous research, *Bacillus* promoted the yield of jujube, corn, rice, and soybean, reduced the toxicity of nitrite in the soil to plants [64,65,66,67], and improved the germination ability of *Spartina densiflora* in heavy metal-polluted areas [68]. *Bacillus* promoted zinc absorption while also inhibiting *Pyricularia oryzae* and *Fusarium moniliforme* in rice [69]. *Sphingomonas* is one of the most effective microbes for cleaning up toxic substances in the soil, and *Sphingomonas* can combine with plants to decompose herbicides [70], promote plant resistance to fungal diseases, and promote plant growth [71]. *Sphingomonas* also has nitrogen fixation and denitrification abilities, which play an important role in maintaining nitrogen balance in nature [72]. *Calcarisporiellomycota* produced β-glucosidase, which is required for the decomposition of cellulose, and was abundant in T2017 [73]. In T2021, *Agaricales* and *Panaeolus* species, which degrade root cellulose and lignin, were present. We inferred that the residual roots of *C. fungigraminus* were able to strengthen microbial communities with stress resistance and disease resistance and confer growth-promoting effects in the sandy soil, providing a good environment for improving the sandy soil and promoting the growth of other plants in the sandy land. These findings suggested that the degradation of plant roots had the potential to regulate the soil microbial community and structural stability, as well as enhance SOM and SOC sequestration after 2020. The plant residual roots increased SMBC and significantly enhanced S-NITS in the rhizosphere sandy soil from 2018 to 2022.

### 4.3. Inspiration of Planting Annual Plants as “Pioneer Plants” in Sandy Land in the Long Term

According to Guo et al., long-term organic fertilizer treatments strongly enhanced CO_2_ emissions and potential mineralized carbon, and these changes in soil properties can be attributed to the variation in microbial communities [74]. Long-term fertilization tracking can also increase soil carbon sequestration in paddy fields and improve nitrogen utilization efficiency in rice [75]. Generally, *Caragana microphylla*, *Haloxylon ammodendron*, *Caragana korshinskii*, *Calligonum mongolicunl,* etc. have become the main plant species selected in artificial sand fixation areas. Annual plants are the first production layer that can cover the surface of sandy soil, and their response and sensitivity to water are extremely high. The growth, survival, and reproduction of annual plants are all affected by the competition intensity of surrounding plants. However, in desert areas, *C. fungigraminus* not only provides a high yield but also leaves a large number of roots in the sandy land after harvest. The results indicated that as the duration of the *C. fungigraminus* residual roots in the sand decreased, the symbiotic relationship between the microbes was enhanced, and the characteristics of the bacterial and fungal co-occurrence network were related to soil physicochemical properties. The changes in the co-occurrence patterns of the residual root rhizosphere sandy soil bacterial and fungal communities were the result of environmental selection. At the same time, the soil physicochemical properties provided sufficient nutrients for fungal colonization, thereby enhancing the connectivity of fungal hyphae. Moreover, plant species affected the soil bacterial diversity and composition. In semiarid ecosystems, changes in the dominant plant species during vegetation restoration efforts have been shown to affect soil bacterial diversity and composition through the direct effects of plants and the indirect effects of soil properties that are driven by plant species [76]. The new species, *C. fungigraminus* was first successfully planted in the Ulan Buh Desert in 2013. In 2018, the residual root system of *C. fungigraminus* reached its fifth year in sandy soil. Extreme temperature and rainy and snowy weather may have led to slow degradation of the residual root length, high moisture, and cellulose content of the plant. *C. fungigraminus,* as an annual herbaceous plant in northwestern China with a high yield, abundant endophytic bacteria, and plant growth-promoting rhizobacteria, can be used as feed and fuel [77,78]. This study further showed that *C. fungigraminus,* as a “Pioneer Plant,” is feasible for desert cultivation and has a positive effect on desert improvement in the long term.

## 5. Conclusions

**Figure 6:**
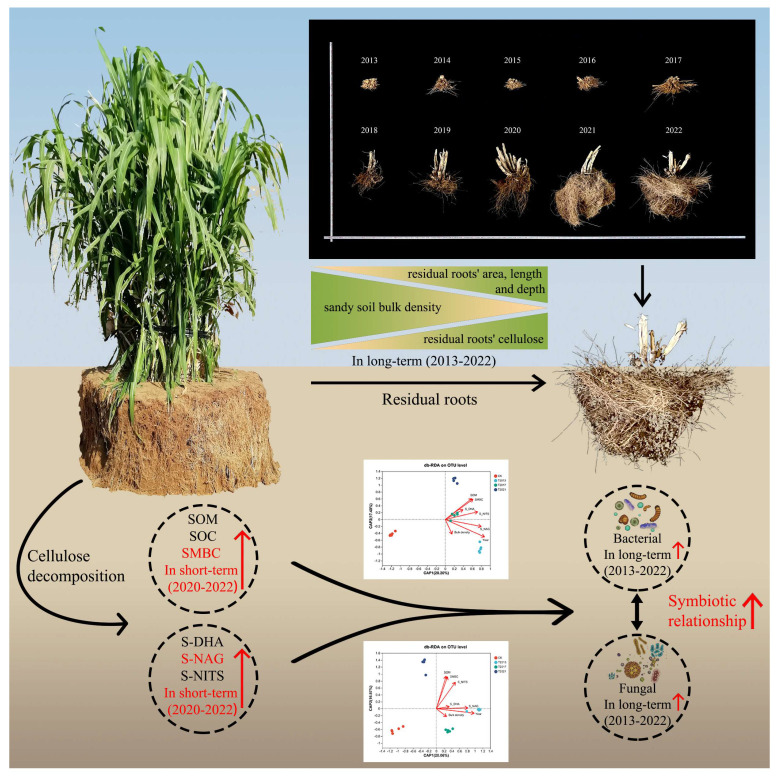
Long-term residual roots of *C. fungigraminus* improve soil SOM, SOC, and SMBC storage and enhance the symbiotic relationship between bacteria and fungi through cellulose decomposition. Note: Soil organic matter (SOM), soil organic carbon (SOC), soil microbial biomass carbon (SMBC), soil dehydrogenase (S-DHA), N-acetyl-β-D-glucosidase (S-NAG), and soil nitrogenase (S-NITS).

We provide new insights into how the residual roots of annual *C. fungigraminus* affect sandy land in the long term in Northwest China. Figure 6 shows that “the residual time-microbial community and diversity-environmental factor” may change over time and suggests that sandy soil fertility, carbon stock, and enzyme activities improved over 3–5 years, while rhizosphere sandy soil diversity and community improved significantly over ten years. Microorganisms degrade residual lignin and cellulose in the root system and transform them to improve soil enzyme activity and microbial diversity in sandy land. Disentangling the plant–microbial–environment tripartite interaction supports more efficient exploitation of plant microbial resources, contributes to predicting the outcomes of global changes in plant–microbial interactions, and develops measures to support “wild-desert” and “desert-farming” ecosystems. Therefore, using annual *C. fungigraminus* as a “Pioneer Plant” to improve sandy soil quality is a new process, providing a theoretical research foundation for the future use of plant and microbial agents to jointly improve sandy soil. However, we will pay more attention to investigating the interaction of traditional sandy plant growth and the *C. fungigraminus* residual roots.

## Figures and Tables

**Table 1:** The physicochemical properties and enzymatic activities of *C. fungigraminus rhizosphere sandy soil* (mean ± standard deviation).

Treatments	CK	T2013	T2014	T2015	T2016	T2017	T2018	T2019	T2020	T2021	T2022
Bulk density (g·cm^−3^)	1.81 ^abcd^ ±0.09	1.86 ^ab^ ±0.06	1.87 ^a^ ±0.03	1.81 ^abcd^ ±0.05	1.84 ^abc^ ±0.07	1.80 ^abcd^ ±0.06	1.79 ^abcd^ ±0.04	1.75 ^cd^ ±0.03	1.81 ^bcd^ ±0.03	1.77 ^abcd^ ±0.08	1.73 ^d^ ±0.04
SOM (g·kg^−1^)	1.79 ^e^ ±0.28	3.15 ^bc^ ±0.43	2.83 ^cd^ ±0.13	2.60 ^cd^ ±0.37	2.54 ^cde^ ±0.28	2.11 ^de^ ±0.23	2.22 ^de^ ±0.30	2.29 ^de^ ±0.27	4.33 ^a^ ±0.81	4.20 ^a^ ±0.48	3.88 ^ab^ ±1.03
SOC (%)	0.10 ^e^ ±0.02	0.18 ^bc^ ±0.02	0.16 ^cd^ ±0.01	0.15 ^cd^ ±0.02	0.12 ^de^ ±0.01^c^	0.12 ^de^ ±0.01	0.13 ^de^ ±0.02	0.13 ^de^ ±0.02	0.25 ^a^ ±0.05	0.24 ^a^ ±0.03	0.22 ^ab^ ±0.06
SMBC (mg·kg^−1^)	89.65 ^e^ ±3.28	107.23 ^bc^ ±5.53	102.68 ^cd^ ±1.91	100.51 ^cd^ ±5.11	99.43 ^cde^ ±4.14	94.68 ^de^ ±3.76	95.37 ^ce^ ±3.86	96.83 ^cde^ ±3.74	121.55 ^a^ ±11.21	119.16 ^a^ ±6.34	117.91 ^ab^ ±14.01
C_t_ (t CO_2_·hm^−2^)	20.66 ^d^ ±3.48	32.79 ^cd^ ±25.3	35.55 ^bcd^ ±8.75	28.06 ^cd^ ±10.7	41.75 ^bcd^ ±23.06	44.91 ^bcd^ ±6.26	73.07 ^b^ ±17.48	63.25 ^bc^ ±12.55	175.53 ^a^ ±20.79	161.01 ^a^ ±27.03	160.00 ^a^ ±28.17
S-DHA(U·g^−1^)	1.88 ^c^ ±1.64	3.18 ^bc^ ±2.51	3.67 ^bc^ ±2.32	4.40 ^bc^ ±1.85	4.37 ^b^ ±2.68^c^	5.70 ^b^ ±1.61^a^	8.43 ^a^ ±3.32	5.78 ^ab^ ±3.89	4.75 ^bc^ ±2.35	4.12 ^bc^ ±2.23	5.95 ^ab^ ±2.04
S-NAG (U·g^−1^)	0.53 ^e^ ±0.09	1.52 ^abcd^ ±0.47	1.81 ^ab^ ±0.63	1.38 ^abcd^ ±0.38	1.27 ^bcd^ ±10.7	1.16 ^bcde^ ±0.32	1.28 ^bcd^ ±0.18	1.73 ^abc^ ±0.67	2.09 ^a^ ±0.41	0.89 ^de^ ±0.26	1.01 ^cde^ ±0.31
S-NITS (IU·L^−1^)	170.58 ^e^ ±9.06	229.85 ^a^ ±10.79	168.27 ^e^ ±12.32	221.45 ^ab^ ±3.41	205.66 ^bc^ ±10.81	175.77 ^de^ ±11.48	218.95 ^ab^ ±8.15	190.36 ^cd^ ±9.08	226.84 ^a^ ±13.37	206.10 ^bc^ ±8.21	229.10 ^a^ ±8.81

Note: Soil organic matter (SOM), soil organic carbon (SOC), soil microbial biomass carbon (SMBC), soil dehydrogenase (S-DHA), N-acetyl-β-D-glucosidase (S-NAG), and soil nitrogenase (S-NITS). Mean values followed by lowercase letters were significantly different (*p* < 0.05).

**Table 2:** The bacterial and fungal community diversity indexes in the rhizosphere sandy soil of the residual roots of *C. fungigraminus* in four treatments (mean ± standard deviation).

Treatments		ACE	Chao	Sobs	Coverage	Shannon	Shannoneven
CK	Bacterial	1315.448 ^c^ ±175.998	1335.925 ^c^ ±175.163	1259.200 ^c^ ±161.788	0.997 ^a^ ±0.001	5.205 ^d^ ±0.057	0.731 ^c^ ±0.011
T2013		3072.304 ^b^ ±318.617	2976.522 ^b^ ±290.213	2400.600 ^b^ ±189.942	0.983 ^b^ ±0.003	5.744 ^c^ ±0.084	0.738 ^c^ ±0.014
T2017		3202.074 ^b^ ±204.347	3152.048 ^b^ ±193.117	2570.600 ^b^ ±136.045	0.983 ^b^ ±0.002	6.092 ^b^ ±0.002	0.776 ^b^ ±0.134
T2021		4368.254 ^a^ ±254.529	4200.932 ^a^ ±248.425	3388.400 ^a^ ±116.999	0.975 ^c^ ±0.003	6.534 ^a^ ±0.081	0.804 ^a^ ±0.008
CK	Fungal	32.366 ^d^ ±4.440	32.000 ^d^ ±4.360	32.000 ^d^ ±4.360	1.000 ^a^ ±0.0001	2.316 ^b^ ±0.230	0.671 ^a^ ±0.070
T2013		116.247 ^c^ ±18.990	119.400 ^c^ ±20.590	108.000 ^c^ ±14.370	0.999 ^b^ ±0.0001	2.537 ^b^ ±0.184	0.544 ^b^ ±0.051
T2017		200.775 ^b^ ±27.470	201.364 ^b^ ±26.900	193.600 ^b^ ±26.300	0.999 ^b^ ±0.0001	3.211 ^a^ ±0.364	0.611 ^ab^ ±0.075
T2021		387.931 ^a^ ±53.300	382.700 ^a^ ±46.896	329.800 ^a^ ±48.122	0.998 ^c^ ±0.001	3.305 ^a^ ±0.222	0.570 ^b^ ±0.027

Note: Mean values followed by lowercase letters were significantly different (*p* < 0.05).

**Table 3:** Topological characteristics of microbial ecological networks under different durations of *C. fungigraminus* residual roots and their corresponding random networks.

Treatments		Degree	Degree Centrality	Closeness Centrality	Betweenness Centrality
CK	Bacterial	691	0.596	0.554	0.259
T2013		756	0.652	0.590	0.202
T2017		826	0.712	0.636	0.270
T2021		909	0.784	0.699	0.379
CK	Fungal	44	0.196	0.386	0.099
T2013		90	0.402	0.458	0.159
T2017		123	0.549	0.530	0.310
T2021		172	0.768	0.689	0.631

## Data Availability

Data will be made available upon request.

## Supplementary Materials

The following supporting information can be downloaded at: https://www.mdpi.com/article/10.3390/plants13050708/s1, Figure S1: Changes in the core species of the residual root rhizosphere sandy soil bacteria (a) and fungi (b) in CK, T2013, T2017 and T2021; Dilution curve of the residual root rhizosphere sandy soil bacteria (c) and fungi (d) in four treatments.; Figure S2: NMDS analysis of the residual root rhizosphere sandy soil bacteria (a) and fungi (b) in CK, T2013, T2017 and T2021. The values of axes 1 and 2 are the percentages that can be explained by the corresponding axes.; Figure S3: Venn map of the residual root rhizosphere sandy soil bacteria (a) and fungi (b) at the phylum level in CK, T2013, T2017 and T2021; The residual root rhizosphere sandy soil bacterial (c) and fungal (d) community and abundance at the phylum level in four treatments; The residual root rhizosphere sandy soil bacterial (e) and fungal (f) species composition map at the genus level in four groups.; Figure S4: LEFse analysis of the co-occurrence correlation of the residual root rhizosphere sandy soil bacterial (a) and fungal (b) communities in CK, T2013, T2017 and T2021, where nodes in the network represent sample nodes or species nodes, and the connection between sample nodes and species nodes represents the inclusion of the species in samples (top 50). The co-occurrence netword analysis on the residual root rhizosphere sandy soil bacterial (a) and fungal (b) communities at genus level showed the co-occurrence relationship of species and different groups.
