# Supplementary figures and images for: Long-Term Benefits of Cenchrus fungigraminus Residual Roots Improved the Quality and Microbial Diversity of Rhizosphere Sandy Soil through Cellulose Degradation in the Ulan Buh Desert, Northwest China
Source: Plants (Basel). 2024 Mar 1;13(5):708. doi: 10.3390/plants13050708 (PMC10935241; doi:10.3390/plants13050708)

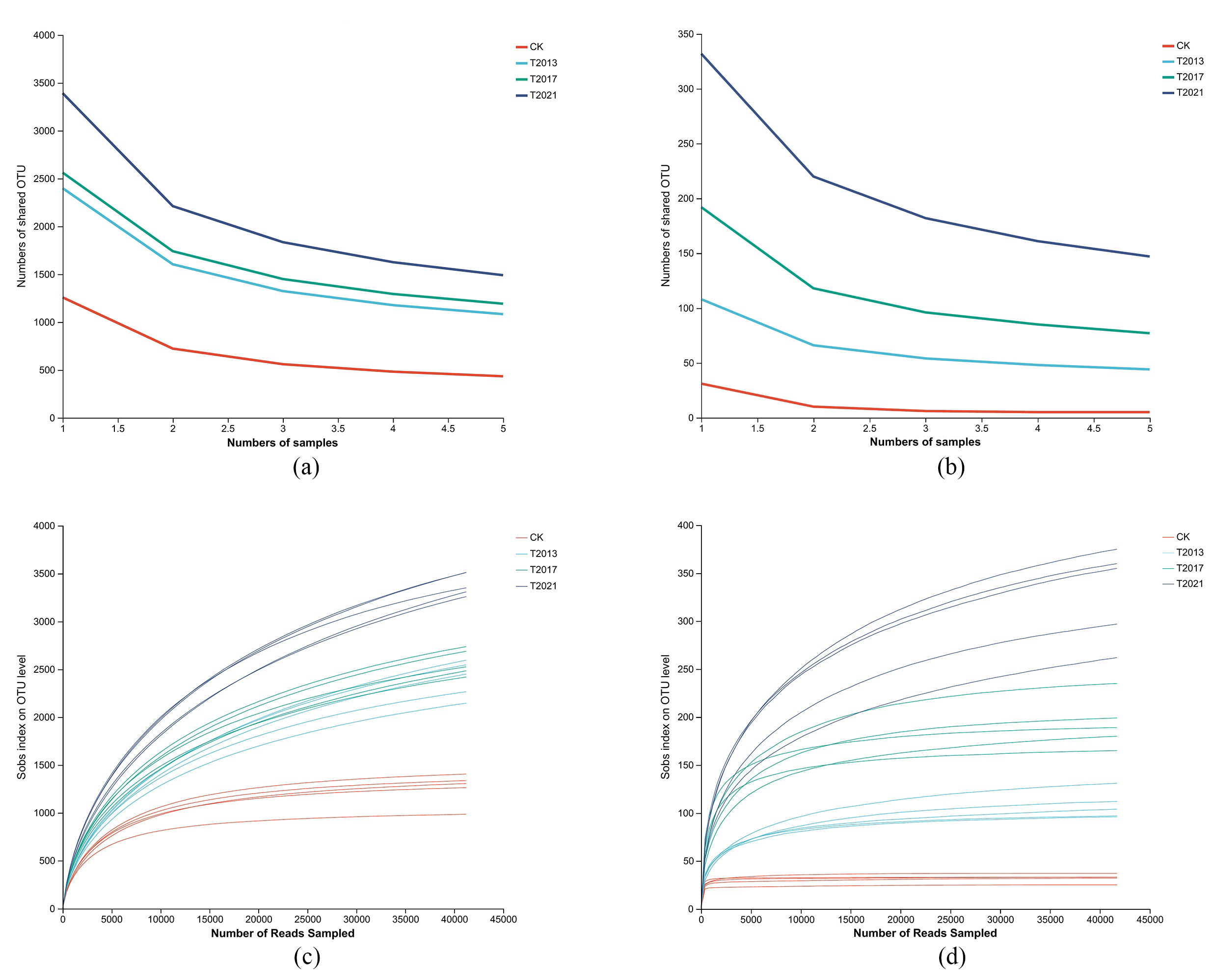

Supplement: Supplementary file 1 [file plants-13-00708-s001.zip › Fig-S1.jpg]

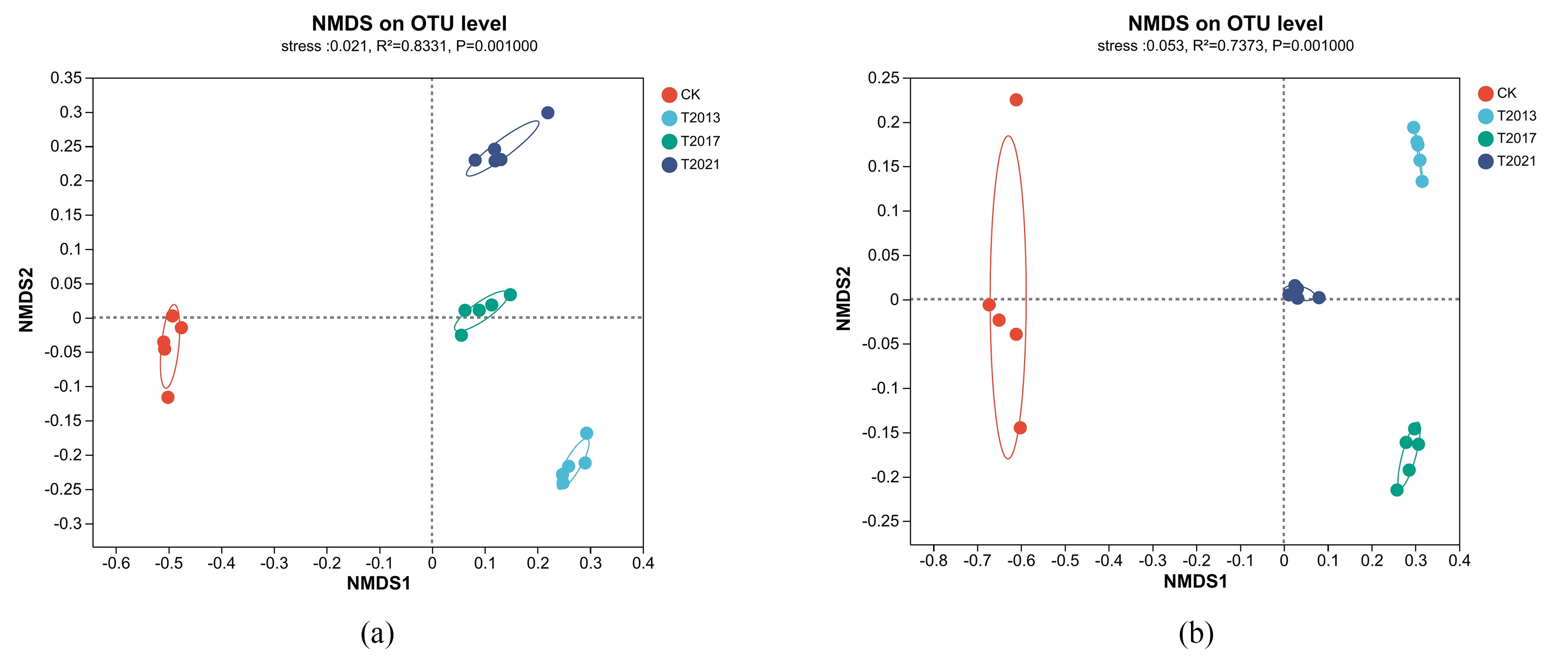

Supplement: Supplementary file 1 [file plants-13-00708-s001.zip › Fig-S2.jpg]

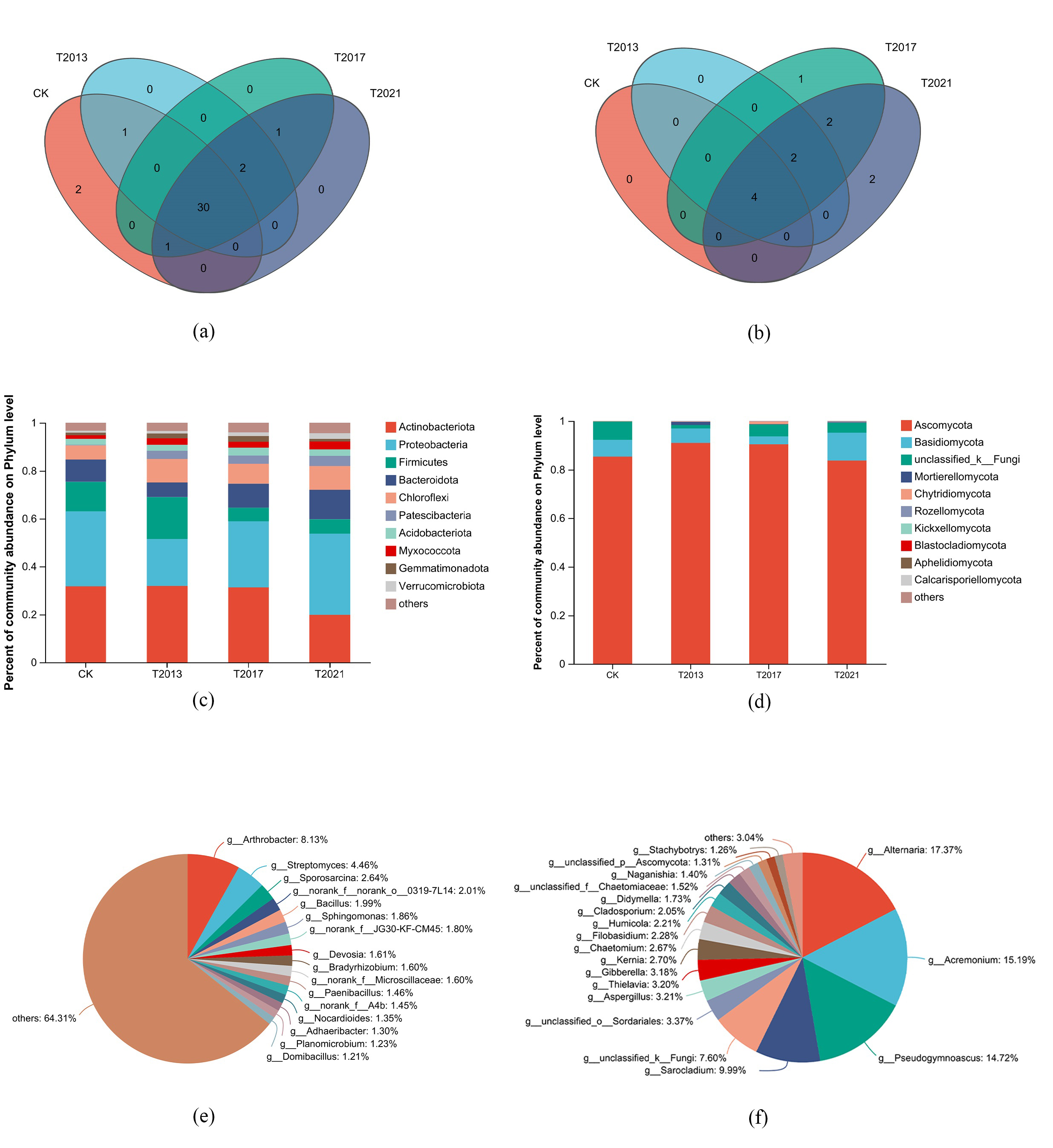

Supplement: Supplementary file 1 [file plants-13-00708-s001.zip › Fig-S3.jpg]

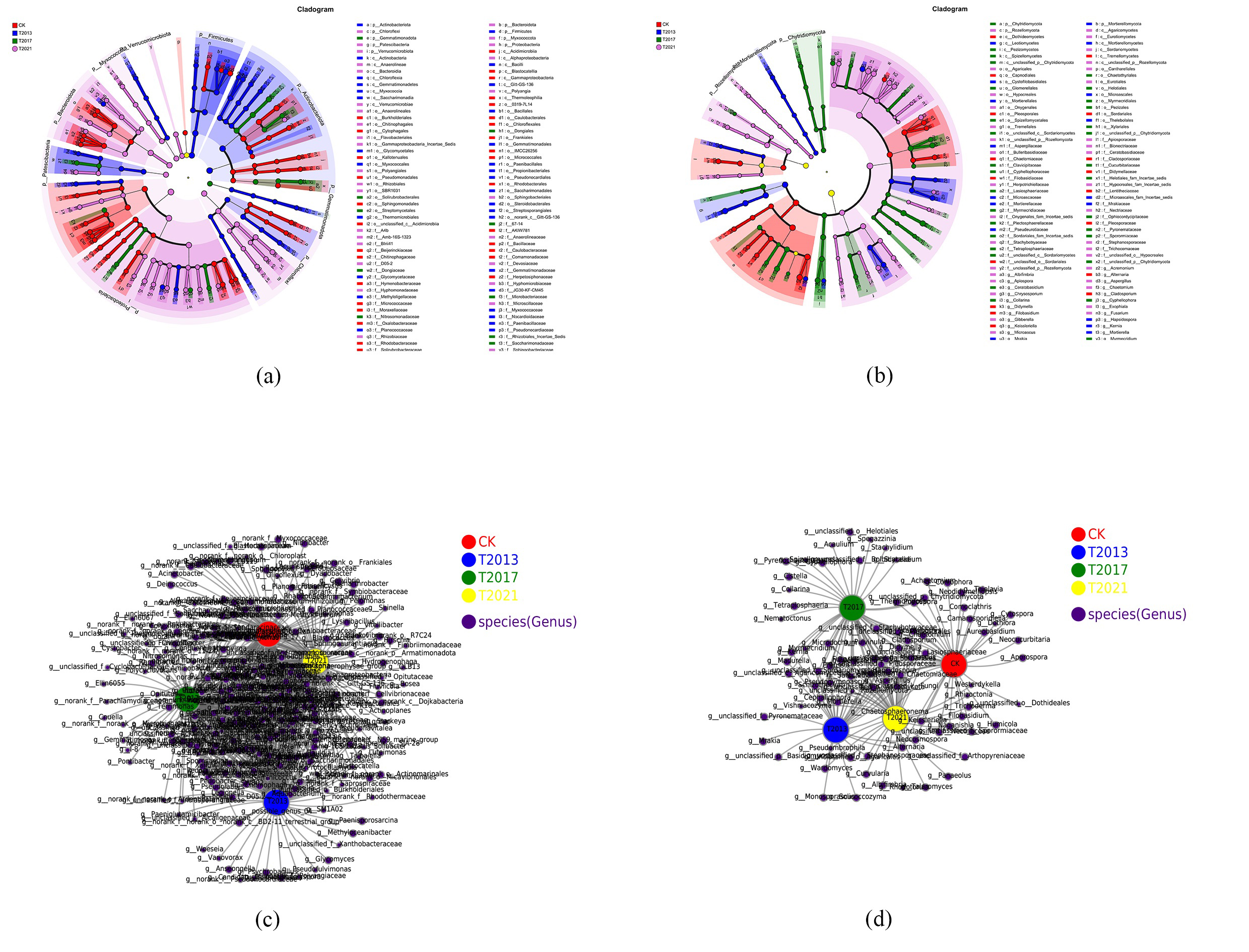

Supplement: Supplementary file 1 [file plants-13-00708-s001.zip › Fig-S4.jpg]
